# A novel dynamic exercise initiative for older people to improve health and well-being: study protocol for a randomised controlled trial

**DOI:** 10.1186/s12877-015-0057-5

**Published:** 2015-06-24

**Authors:** Myrla Patricia Reis Sales, Remco Polman, Keith D. Hill, Tuire Karaharju-Huisman, Pazit Levinger

**Affiliations:** Institute of Sport, Exercise & Active Living (ISEAL), College of Sport and Exercise Science, Victoria University, PO Box 14428, Melbourne, VIC 8001 Australia; Department of Psychology, Bournemouth University, Poole House P104a, Talbot Campus, Fern Barrow, Poole, BH12 5BB UK; School of Physiotherapy and Exercise Science, Curtin University, GPO Box U1987, Perth Western, 6845 Australia

**Keywords:** Falls prevention, Older adults, Exercise park, Social interaction, Effectiveness

## Abstract

**Background:**

Exercise is an important and effective approach to preventing falls in older people, but adherence to exercise participation remains a persistent problem. A unique purpose-built exercise park was designed to provide a fun but physically challenging environment to support exercise in a community setting. This project is a randomised controlled trial designed to evaluate the effectiveness of an exercise intervention using an exercise park specifically designed for older people in reducing the risk of falls.

**Methods/Design:**

This study will be a parallel randomised control trial with pre and post intervention design. One hundred and twenty people aged between 60 and 90 years old will be recruited from Melbourne suburbs and will be randomly allocated to either an exercise park intervention group (EPIG) or a control group (CG). The CG will receive social activities and an educational booklet on falls prevention. The BOOMER balance test will be used as the primary outcome measure. Secondary outcome measures will include hand grip strength, two minute walk test, lower limb strength test, spatio-temporal walking parameters, health related quality of life, feasibility, adherence, safety, and a number of other psychosocial measures. Outcome assessment will be conducted at baseline and at 18 and 26 weeks after intervention commencement. Participants will inform their falls and physical activity history for a 12-month period via monthly calendars. Mixed linear modelling incorporating intervention and control groups at the baseline and two follow up time points (18 weeks and 26 weeks after intervention commencement) will be used to assess outcomes.

**Discussion:**

This planned trial will be the first to provide evidence if the exercise park can improve functional and physiological health, psychological and well-being. In addition, this study will provide empirical evidence for effectiveness and explore the barriers to participation and the acceptability of the senior exercise park in the Australian older community.

**Trial registration:**

This trial is registered with the Australian New Zealand Clinical Trials Registry - Registry No. ACTRN12614000700639 registered on Jul 3rd 2014.

**Electronic supplementary material:**

The online version of this article (doi:10.1186/s12877-015-0057-5) contains supplementary material, which is available to authorized users.

## Background

Falls are a leading cause of death and disability among older adults [1]. About one third of people aged 65 years or older fall at least once a year [1, 2]. In 2013, Australia had 14 % of its population aged 65 years and over [3] with approximately 10 % have multiple falls [4] and 20 % of those who fall experience injuries requiring medical attention [2]. In 2011–12, there were 88,386 fall related hospitalisations in Australia for people aged over 65 years, with the most common injury being hip or other lower limb fracture [5]. Hip fractures in particular are associated with high level of mortality and morbidity, with recent studies reporting that 20 % of hip fracture patients die within 12 months of injury [6], and over half do not regain pre-fracture mobility or large muscle group abilities up to two years after the fracture [7].

Most falls are associated with one or more identifiable risk factors [8] and the risk of falling has a direct association with the number of risk factors involved [9]. Physiological factors, such as lower extremity muscle weakness, gait and balance impairments and functional impairments, have been highly associated with the risk of falls and are most often targeted by preventive programmes [9]. Therefore, targeting these modifiable risk factors through exercise programmes seems to be a suitable way to reduce the risk of falls [2, 10].

Exercise programmes have been shown to be effective in reducing the risk of falling and the rate of falls [10] as they can improve muscle strength, flexibility, balance, coordination, proprioception, reaction time and gait [11]. These positive outcomes have been observed even in the very old and frail [11]. A meta-analysis identified that 50 h cumulative exercise (irrespective of exercise frequency) is needed to reduce the risk of falls [12]. A recent telephone survey applied in NSW has shown that older people’s participation to strength or balance-challenging activities was 21.0 % (95 % CI: 9.8–22.2) with only 5.3 % participating in both forms (strength and balance-challenging activities) [13]. Thus, there is a need to improve long-term participation in physical activity, which is not a common habit for most older individuals [14].

The “exercise park for older people” was originally introduced in Europe in 2009 as a novel purpose-built exercise park designed to improve muscular strength, flexibility, coordination and balance through active fun activities. The exercise park aims to inspire older people to be playful, to exercise, and to challenge their bodies, which can lead to a more active and healthier life style. Such indoor and outdoor exercise parks are widely available in Finland, Spain and China with only few operating in Australia. Preliminary data from The Netherlands suggests the purpose-built exercise parks may be safe and acceptable to older people. However, no evidence-based research exists that demonstrates the effectiveness of the exercise park in improving physical health, psychological well-being or independence. A report from The Netherlands [15] provided preliminary evidence of the feasibility of a 10 weeks exercise program with the exercise park equipment for a small sample of older people (*n* = 13), reporting high attendance (92 %), reduction in fear of falling and increased muscle strength and balance. Although these results are promising, further research is needed with a larger sample size and a longer intervention period to determine the effectiveness of the exercise park program on physical and psychological outcomes, well-being and independence of older adults, as well as determining the feasibility and acceptability of this type of program in the Australian community.

Therefore, the aims of this study are (1) to evaluate the effectiveness of such an exercise park in reducing the risk of falls and (2) to evaluate what other benefits, including psychosocial, functional and physiological, can be achieved by using this specific exercise park for 18 weeks with a structured and progressive program in community dwelling older adults using a randomised controlled trial design and (3) what benefits are sustained eight weeks after completion of the exercise park program.

## Methods and design

All procedures involved in this trial will be conducted in compliance with National Statement on Ethical Human Resource and the Australian Code for the Responsible Conduct of Research. Ethical approval has been obtained from the Human Research Ethics Committee from Victoria University, Melbourne (Application ID. HRE13-215). The study was design according to the Consolidated Standard of Reporting Trials (CONSORT) guidelines and publications associated with the trial will be reported according the CONSORT 2010 Statement [16, 17].

### Design and setting

This study will be a parallel randomised controlled trial (RCT) with pre and post intervention design (outcome assessments at baseline and at 18 and 26 weeks after participation commencement) comparing an exercise park intervention program for older people with a control group, aiming at evaluating the effectiveness of an exercise intervention using an exercise park specifically designed for older people in reducing the risk of falls.

### Participants

One hundred and twenty older people living in the community aged between 60 and 90 years old who have had one or more falls in the previous 12 months or who are concerned about having a fall will be recruited. Participants who are generally active and independent in the community with no more than a single point stick used for regular outdoors walking (at least three times per week) will be included. The aim in these inclusion criteria is to target those with mild falls risk, but who remain relatively active, using a health promotion and prevention approach.

Older adults will be excluded from this study if they have: 1) any uncontrolled non-musculoskeletal conditions that would make testing difficult and uncomfortable, such as chronic obstructive airways disease and congestive heart failure; 2) a pre-existing neurological or orthopaedic condition that affects lower limb strength (e.g.: polio, stroke); 3) any of the following foot conditions: partial foot amputation or ulceration or foot fractures; 4) any uncontrolled musculoskeletal conditions that may affect ambulation (rheumatoid arthritis, gout, etc.). Participants with heart problems (e.g. chest pain (angina), heart murmur, heart rhythm disturbance, heart valve disease or heart failure) will be required to obtain a medical clearance from their general practitioner in order to participate in this study. Participants with any documented medical condition or physical impairment that is judged by the medical practitioner to contraindicate their inclusion will be excluded. Written informed consent will be sought from the participants

### Recruitment and randomization

**Fig. 1:**
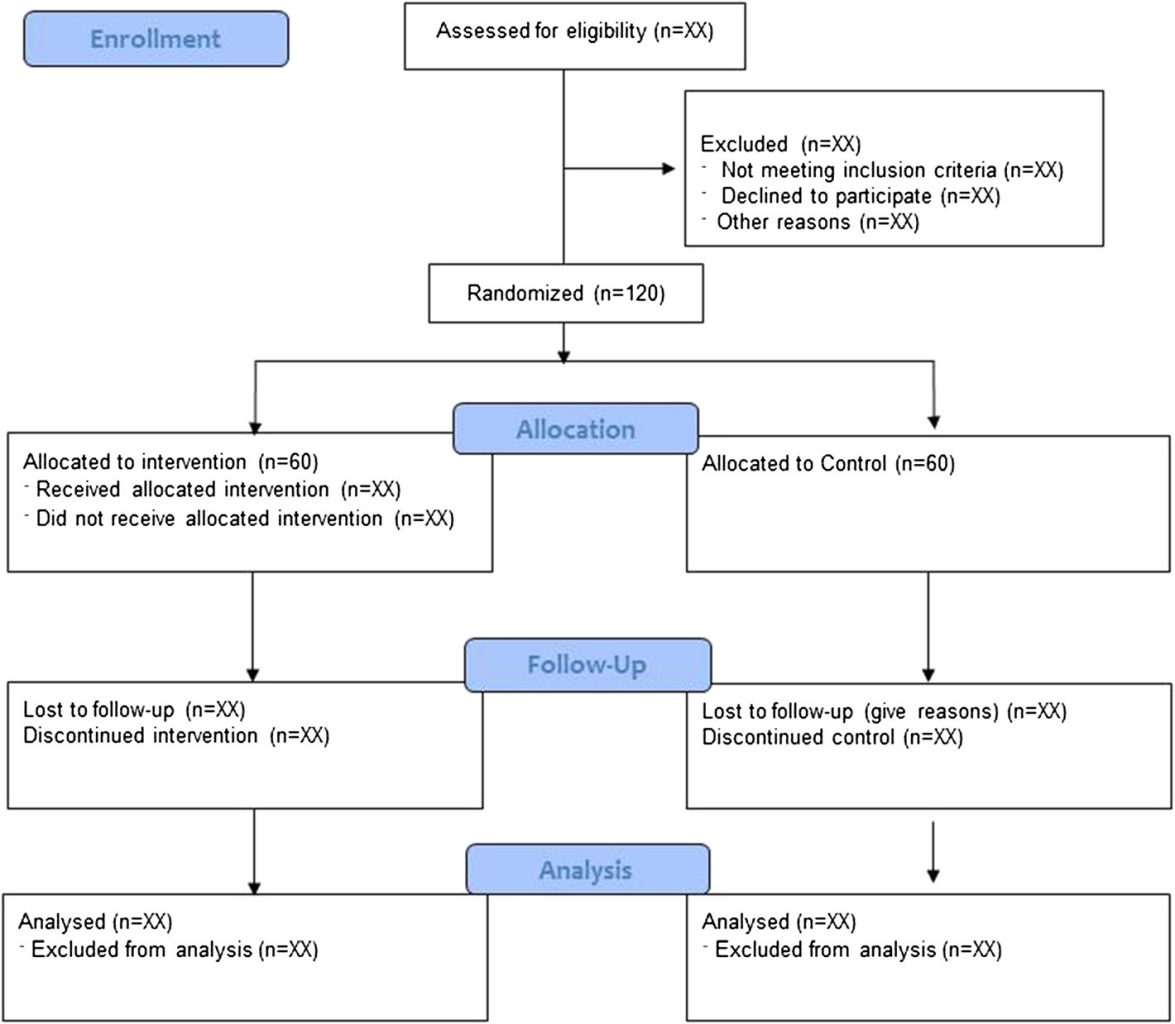
Consort flow diagram of recruitment and randomization

Participants will be recruited from Melbourne suburbs. Local senior organizations, retirement villages, community centres, senior clubs and associations in the areas around the park location will be contacted for recruitment purposes. Participants will be also recruited via community health promotion events and advertisement in local newspapers, magazines and online social networking media. Participants will be informed about the project by posters placed in healthcare facilities and places with high circulation of senior citizens and mail-out advertisements to health care practitioners in Melbourne. Participants will be randomly allocated to one of the following groups: (1) Exercise Park Intervention Group (EPIG) or (2) Control Group (CG). Block randomization stratification by gender will be undertaken, so that blocks of 12 participants will be recruited at a time, randomized into one control group of six participants and one exercise groups of six participants (Fig. 1). To accommodate couples (e.g. partners/married couples) participation, randomisation by couple will also take place. Assessors will prepare the envelopes with six paper codes (three exercise intervention and three control group) which will be added to opaque not concealed envelopes. There will be three envelopes: one for couples, one for females and one for males. Participants will be asked to pick one paper from their respective envelope and the picked paper will assign the participant to either the exercise intervention group or control group. Recruitment will be undertaken over a period of 14 months to achieve a sample size of 60 participants in each group. Assessors and participants will not be blinded to their respective group allocation (EPIG or CG).

Participants from the EPIG will undergo an 18-week exercise intervention. The exercise sessions will be provided two times a week (each class approximately 1 to 1.5 h duration) and will be supervised by a qualified physiotherapist or an accredited exercise physiologist. Each session will consist of 5 to 10 min warm-up exercises, followed by 45 to 75 min on the equipment stations, and will conclude with 5 to 10 min of cool down exercises. The exercise classes will include 6 to 8 participants and will be circuit-based with the warm up and cool down exercises being performed in a group and the core session being carried out in training pairs. Participants will be performing exercises that focus on strength, balance, coordination, mobility and flexibility as detailed in Tables 1, 2, 3, 4 and 5. Exercisers will be paired in stations and an exercise session can include up to eight stations (See Table 6). The intervention program will be carried out at St Bernadette’s Community Respite House, with no cost to the participants.

**Table 1:** Strength exercises to be performed using the senior exercise park with their respective levels of progression

Exercise	Description	Functional relevance	Progressions
Push-up bar	Participant pushes body up away from the bar and brings it down towards the bar.	Strengthens arms, back and core muscles.	Standing nearly perpendicular to the bar:
			Level 1 – Wide grip.
			Level 2 – Narrow grip.
			Level 3 – Wide grip standing on a 10cm high block*.
			Level 4 – Narrow grip, standing on a 10cm high block*
			Level 6 – Narrow grip, hand release.
			Level 7 – Narrow grip, front knee tucks.
			Level 8 – Narrow grip side knee tucks.
			Level 9 - Perform the push-ups with 1 hand. Hand on shoulder line.
Modified Pull-Ups	Participant pulls body up towards the bar.	Strengthens arms, back and core muscles.	Level 1–3 – Hands narrow (undergrip), increase distance from the bar (3 distances 3 distances determined by a line on the floor).
			Level 4–6 – Hands wide (undergrip) – increase distance from the bar (3 distances)
			Level 7–9 – Hands narrow (overgrip) – increase distance from the bar (3 distances).
			Level 10–12 – Hands wide (overgrip) – increase distance from the bar (3 distances).
			If the participant reaches RPE 4/10 again, the wooden 10-cm high block can be introduced on the exercise and all levels are repeated again.
Calf Raises	Participant raises the heels until the body is on tiptoes to work the calf muscles and, at the same time, climbs the finger steps to reach the highest point possible.	Important for stability, posture and mobility as well as help the blood circulation.	Level 1 – Facing the bar, double leg heel raise, 2 hands
			Level 2 – Facing the bar, single leg heel raise, 2 hands
			Level 3 – Side on to the bar, double leg heel raise, 1 hand
			Level 4 – Side on to the bar, single leg (standing on outermost) heel raise, 1 hand
			Level 5 – Side on to the bar, single leg (standing on innermost) heel raise, 1 hand
Bar – Hip Extension	Participant, with control and keeping back and knee straight and foot flexed, slowly take leg backwards tightening bottom muscles.	Strengthens gluteal and hamstrings muscles and works balance.	Alternating legs, 5 on each:
			Level 1 – Comfortable speed
			Level 2 – Pulse twice at the top part of the movement.
			Alternating legs, 10 on each:
			Level 3 – Comfortable speed
			Level 4 – Pulse twice at the top part of the movement.
			Alternating legs, 15 on each:
			Level 5 – Comfortable speed
			Level 6 – Pulse twice at the top part of the movement.
Step-ups	Participant steps up and down the platform.	Improves ability for using stairs and getting in and out the bath or bus.	Level 1 – Alternating legs, with hand support.
			Level 2 – Alternating legs, no hand support.
			Level 3 – 5 on each leg, with hand support.
			Level 4 – 5 on each leg, no hand support.
			Level 5 – 10 on each leg, no hand support.
			Level 6 – Sideways, 5 on each leg, no hand support.
			The 10cm high wooden block can be introduced before each level if participant reports a RPE greater than 7/10.
Bar – Hip Abduction	Participant moves their leg to side with straight knee.	Strengthens hip stabilizer muscles and works balance.	Level 1 – Comfortable speed, 5 repetitions, alternate legs.
			Level 2 – Pulse twice at the top of the movement, 5 repetitions, alternate legs.
			Level 3 – Comfortable speed, 10 repetitions, alternate legs
			Level 4 – Pulse twice at the top part of the movement, 10 repetitions, alternate legs.
			Level 5 – Comfortable speed, 15 repetitions, alternate legs.
			Level 6 – Comfortable speed, 20 repetitions, alternate legs

*Wooden block dimensions: L 70cm x W 40cm x H 10cm

**Table 2:** Balance exercises to be performed using the senior exercise park with their respective levels of progression

Exercise	Description	Functional relevance	Progressions
Gangway	Participant walks along the rickety bridge surface.	Helps to find a good stance for uneven and unstable surfaces like on the bus or underground.	Level 1 – 2 hands support, 1 foot per step.
			Level 2 – 1 hand support, 1 foot per step.
			Level 3 – no hand support, 1 foot per step.
Balance stool	Balancing on an unstable stool.	Exercises the deep muscles that support the spine.	Level 1 – Pushing down edges of the stool, 2 hands on the bar.
			Level 2 – Pushing down edges of the stool, 1 hand support.
			Level 3 – Pushing down edges of the stool, no hand support.
			Level 4 – Pushing down edges of the stool, hands overhead.
			Level 5 – Pushing down edges of the stool, alternating hands overhead.
Balance Beam	Participant walks back and forth along the beam.	Improves walking safely on awkward surfaces such as natural and unpaved paths. Walking on an undulating balance beam is a good balancing exercise.	Level 1 – 1 hand for support, normal walking.
			Level 2 – Heel to toe walking, hand support.
			Level 3 – Heel to toe walking, no hand support.
			Level 4 – Walking on toes with hand support.
			Level 5 – Walking on toes with no hand support.
			Level 4 – Normal walking with semi-squat, hand support.
			Level 5 - Normal walking with semi-squat, no hand support.
			Level 6 – Cognitive dual-task counting down by 2 and no hand support.
Ramp + Net Walking Through Walking on the ropes	Participant walks up the ramp and steps down either through the net or on to the ropes, climbs through under the bar and walks back on heels and toes to the ramp.	Strengthens and exercises the lower limbs.	Walking through the net without hitting the ropes:
		Improves spatial awareness and coordination.	Level 1–3 – Narrow stance, ranging from 2 hand support, 1 hand support and no hand support
		Improves balance.	Level 4–6 – Wide stance, ranging from 2 hand support, 1 hand support and no hand support
			Walking balancing on the ropes:
			Level 7–9 – Narrow stance, ranging from 2 hand support, 1 hand support and no hand support
			Level 10 – On crosses of netting, no hand support
			Level 11–13 – Wide stance, ranging from 2 hand support, 1 hand support and no hand support
			Participant alternates the way he/she comes up the ramp by walking on toes or on heels.
			After reaching level 13, participant can come back to the ramp doing lunges.
			If ramp is too high for participant to come down before walking through the net, a wooden block (L 70cm x W 40cm x H 10cm) can be introduced until participants improves level of conditioning, strength and balance.

**Table 3:** Coordination exercises to be performed using the senior exercise park with their respective levels of progression

Exercise	Description	Functional relevance	Progressions
Taps on Platform	Participant taps on the platform with feet.	Improves ability for using stairs and getting in and out the bath or bus.	Level 1 – Taps on the platform, alternating legs, hand support
			Level 2 – Taps on the platform, alternating legs, arms in front of the body.
			Level 3 – Taps on the platform, alternating legs, arms above head.
			Being the platform too high for participant to tap, a wooden block (L 70cm x W 40cm x H 10cm) can be introduced until participants improves level of conditioning, flexibility, strength and balance.

**Table 4:** Flexibility and mobility exercises to be performed using the senior exercise park with their respective levels of progression

Exercise	Description	Functional relevance	Progressions
Rounded Snake Pipe	Participant moves the ring from one end to the other without touching the bar.	Strengthens and mobilises the shoulders.	Level 1 – Side facing, walking, and looking forward.
		Improves hand–eye coordination and concentration skills.	Level 2 – Side facing, walking on heels and toes, looking forward.
		Helps getting dressed, combing hair, washing oneself, hanging up clothes.	
Sharp Snake Pipe	Participant stands on mark and moves the ring from one end to the other without touching the bar.	Improves balance, trunk mobility and abdominal muscle strength.	Facing the snake pipe:
			Level 1 – Feet together, change hands in the middle.
			Level 2 – Feet together, same hand reaching across the body
			Side on to the snake pipe:
			Level 3 – Feet together, reaching forward, 5 each side.
			Level 4 – Feet together, reaching forward and backward, 5 each side.
			Level 5 – Feet together, reaching forward and backward, one side per set.
			Level 6–7 – Standing on one leg (outermost), ranging from 5 repetitions to 10 repetitions on each side.
			Level 8 – Standing on one leg (outermost), one side per set
			Level 9–10 – Standing on one leg (innermost), ranging from 5 repetitions to 10 repetitions on each side.
			Level 11 – Standing on one leg (innermost), one side per set.

**Table 5:** Functional exercises to be performed using the senior exercise park with their respective levels of progression

Exercise	Description	Functional relevance	Progressions
Screw and Turner	Participant turns the screw and turner each direction whilst standing on one leg.	Improves daily activities such as opening doors and taps.	Level 1–3 - Single leg stance (SLS), ranging from 5, 10 and 15 repetitions each direction, so alternate legs.
		Helps with opening doors and jars.	Level 4 – SLS, 15 repetitions each direction. Same leg for the whole set.
			Level 5 – SLS, 20 repetitions each direction. Same leg for the whole set.
Sit to Stand	Participant sits and stands up from the seat or stands to squat and touch the bench.	Strength of muscles on lower limb and balance.	Not using the 10cm high block*:
			Level 1 – Sit to stand (STS) with hand support
			Level 2 – STS with arms in front of the body.
			Level 3 – STS with arms crossed on the chest.
			Using the 10cm high Block*:
			Level 4 – STS with hand support.
			Level 5 – STS with arms in front of the body.
			Level 6 – STS with arms crossed on the chest.
			Level 7 – Squatting to touch the bench, arms in front of the body.
			Level 8 – Squatting to touch the bench, bench arms crossed on the chest.
			Not using the 10cm high block*:
			Level 9 – STS pushing off with 1 leg mostly and lifting heel, alternating legs.
			Level 10 – STS pushing off with 1 leg mostly and lifting foot from the floor, alternating legs, with hand support.
			Level 11 – STS pushing off with 1 leg mostly and lifting foot from the floor, alternating legs, arms crossed on the chest.
			Level 12 – Sit to stand pushing off with 1 leg mostly and lifting foot from the floor, 5 on each, arms crossed on the chest.
			Add the 10cm high block*, participants repeats the same progression from level 10 to level 12.
Stairs	Participant steps up and down the steps.	Strengthens the heart and lower limbs.	Level 1–3 – stepping up and down slowly ranging from 2 hands for support, 1 hand for support and no hand support.
	The handrail makes the exercise safe.		Level 4–6 - stepping up every second step ranging from 2 hands for support, 1 hand for support and no hand support.
	Steps can also be used for stretching exercises.		

*Wooden block dimensions: L 70 cm x W 40 cm x H 10 cm

Abbreviation: STS = sit to stand

**Table 6:** Exercise stations

Station number	Exercise 1	Exercise 2
1	Push-ups	Taps on Platform
2	Modified Pull-ups	Gangway
3	Balance Stool	Calf Raises + Finger Steps
4	Sit to Stand	Rounded Snake Pipe
5	Ramp + Net + Climb Through	Sharp Snake Pipe
6	Balance Beam	Hip extension
7	Stairs	Screws / Turners
8	Step-ups	Hip Abduction

Participants in the CG will be advised to continue with their usual daily activities and will be meeting the research team every two weeks to take part in some social activities (nine meetings of two hours duration over 18 weeks of intervention). Participants from both groups will be tested at the following timelines: baseline, at the end of the intervention period (18 weeks) and two months after that (26 weeks after intervention commencement).

### Treatment preference and credibility/expectation

Research has shown that participants who are allocated to their preferred treatment achieve better outcomes than those who do not receive their preferred treatment, despite the randomisation process resulting in equivalent baseline outcome measure scores [18]. To address this issue, participants will be asked if they have a preference for one of the two groups they can be allocated to (documented as control group, exercise intervention group or no preference). However, their response will not influence their randomised group allocation [19]. It is expected that this approach conserves all the advantages of a randomised design. In addition, it enables the interaction between preference of participants and outcomes to be quantified in later stage of analyses [20].

### Outcome measures

Socio-Demographic factors (such as age, gender, education and previous occupation), medical conditions, medications currently prescribed, main surgeries and medical procedures undergone, smoking habits as well as alcohol consumption will be obtained via a structured questionnaire. Anthropometry will include body weight and height. Height and weight will be measured using a stadiometer and digital scales respectively, and body mass index will be calculated as weight (kg)/height (m^2^).

#### Primary outcome: the Balance Outcome Measure for Elder Rehabilitation (BOOMER)

Due to the importance of balance in preventing falls, and given that balance is multi-dimensional, a test battery that incorporates a number of key domains of balance (static and dynamic balance, including measures of stepping, reaching and turning, that are commonly involved in falls) will be used as the primary outcome to assess the effectiveness of this novel purpose-built exercise park in improving several physiological, biomechanical and psychosocial factors associated with the risk of falls. The Balance Outcome Measure for Elder Rehabilitation (BOOMER) is a multi-item balance measure, which comprises 4 well validated clinical measures (step test [21], timed up and go (TUG) [22], functional reach (FRT) [23], and static standing balance [24–26], and will be used as the primary outcome measure. The four individual components of the BOOMER can be scored individually or as a composite score and will be described as follows:

- Functional Reach Test [23]—the participant will be asked to stand next to a white board with feet hip width apart and closed fist, and extend their dominant arm horizontally at approximately 90° then reach as far as possible without taking a step or losing their balance. Lines will be drawn to mark the initial position of the participant’s arm (zero position) and the reach forward position. The difference between the two marks will be measured [23]. There will be no attempt to control the participant’s method of reach apart from making sure that participant is not twisting their body to achieve a further reach. Each participant will be given one practice trial and the best of two test trials will be used for the assessment.
- Static Balance Standing—For the static timed standing with eyes closed and feet together test, the participant will be asked to stand still on the floor with shoes on and eyes closed. The result will be recorded as a sum of three trials on which the participant can stand on this position. However, if the first trial records the maximum score of 30 s, then the subsequent two trials will be automatically scored as 30 s as per original procedure [24, 27].
- Step test—The step test is a measure of a dynamic single limb stance task [21]. Using a 7.5-cm high block, the participant will be asked to place his/her foot onto the top of and back to the floor as many times as possible in 15 s [21]. Participants will be given time to practice (around two correct cycle of steps) and one formal trial will be performed on the dominant leg.
- Timed Up and Go test—The Timed Up and Go test is a dynamic and functional performance measure of overall mobility, and balance [28, 29]. This test also evaluates the ability of an individual to turn 180° while maintaining the upright position and the ability to maintain the upright standing position immediately after transition from a seated posture [29]. The participant will be instructed to stand from a standard 43 cm high armless chair, walk to a cone placed 3 m away from the chair, turn around the cone and return back to the chair and sit. Participants will be asked to perform one practice trial and four testing trials. The testing trials will include two comfortable/preferred speed [29] and two fast speed Timed Up and Go tests [30]. The best time of each of the two speeds will be used for analysis. However, to compose the BOOMER measure, only the comfortable speed trial will be used. Participants will be allowed to use a gait aid if one is used routinely for indoors walking.

#### Secondary measures

The following functional tasks and psychosocial variables will be assessed:

1. Hand grip strength test [31] will be used to measure muscle strength. Hand-grip strength is a simple, reliable, inexpensive surrogate of overall muscle strength and a valid predictor of physical disability and mobility limitation [32]. Using a TTM digital hand dynamometer (Mentone Educational Centre, Melbourne, VIC), participants will be asked to perform two maximum force trials with each hand and the best score of two attempts will be recorded. Participant will be seated on a 43 cm high chair, feet flat on the floor, with shoulder adducted and neutrally rotated, elbow flexed at 90° and forearm in neutral and the wrist between 0 and 30° extension and between 0° and 15° ulnar deviation [33]. The maximum values of the left- and right-hand grip measurements will be summed and be used for the analysis to remove consideration of hand dominance [31].
2. Two minute walk test will be used to assess exercise tolerance [34] and functional mobility [35]. Improvement in distance walked within the test interval is attributed to improvement in cardiac output, in mechanics of ventilation, or in muscular conditioning [36]. Participants will be asked to walk for 2 min on a demarcated area at a comfortable pace and the maximum distance achieved will be recorded. Participant will be allowed to use their gait aid if regularly used for indoors walking.
3. Lower limb strength will be assessed via the sit-to-stand test [37] and measurement of the strength of the knee extensor muscles using a purposely built force transducer [38]. The sit to stand test is a simple test used to measure mobility and lower limb strength [37] and is also included in fall risk assessments [39, 40]. Participants will be asked to stand from a 43 cm high chair as many times as possible for a period of 30 s without any assistance of the assessor. Participants will be asked if they need their hands to assist them in standing up from the chair and this information will be recorded for further analysis. Otherwise, arms will be kept to the side of their body during the test.
4. The strength of the knee extensor muscles of both limbs will be measured with a purposely built force transducer which will be attached to the participant’s leg using a webbing strap with a Velcro fastener. The participant will sit on a tall chair with a strap around the lower leg 10 cm above the ankle joint, and the hip and knee joint angles will be positioned at 90°. The distance from the knee joint to the strap around the ankle will be measured with a tape measure. This measure will be used for the calculation of torque (i.e. force [N] distance [m]). The maximum voluntary contraction will be assessed during an isometric knee extension. Participants will be asked to perform three maximum voluntary contractions trials for each leg. The contractions will last up to five seconds each, with a rest period of one minute between each trial. The force data will be stored on a portable computer. The best performance of the three trials will be considered as the maximum torque for each side.
5. Spatio-Temporal Gait Parameters: Measures of stride dynamics and gait variability have been shown to identify fallers in older adults with gait limitations and those with a history of falls [41, 42]. Assessment of walking speed, stride length, stride width and double limb support will be performed with the use of the GaitRite® system (CIR System, Inc, Harverton PA) instrumented walkway system (active length of the mat: 8.75 m). Participants will be asked to start from a point 3 m in front of the mat and will stop on a point 3 m behind the mat. Approximately 10 strides per participant are required to achieve reliable mean estimates of spatio-temporal gait parameters including velocity, stride and step length, and step and single support time [43, 44]. Therefore, seven walks will be recorded to allow sufficient data to be collected. Multiple practice trials will be given until participants feel comfortable and will be walking with consistent velocity. This will be followed by seven testing trials which will allow sufficient number of strides to be recorded. Participants who use a gait aid for indoors walking will be allowed to use it during the tests. Participants will be wearing flat shoes during the test.
6. The following questionnaires will be used to evaluate health related quality of life measures and psychological or psychosocial measures:
  - *The Short Form (12) Health Survey Version 2 (SF-12v2™)* is a 12-item questionnaire which evaluates the individual health status over eight domains including vitality, physical functioning, bodily pain, general health perceptions, physical role functioning, emotional role functioning, social role functioning, and mental health [45]. The SF-12v2*™* has been given preference for use among older people (compared with other longer versions of measures of quality of life such as SF-36) because of its brevity. [46]. Most questions use a five-value response option (all of the time, most of the time, some of the time, a little of the time, and none of the time) and some a three-value response option (yes, limited a lot, limited a little or not limited at all). Physical and Mental Health Composite Scores (PCS & MCS) are computed using the scores of twelve questions and range from 0 to 100, where a zero score indicates the lowest level of health measured by the scales and 100 indicates the highest level of health [45].
  - *The Incidental And Planned Activity Questionnaire (IPAQ)* for older people will be used to assess the physical activity level of the participants [47]. The IPAQ is a self-report questionnaire that covers the frequency and duration of several levels of planned and incidental physical activity in older people. Planned activities (6-items) include planned exercise or walks whereas incidental physical activities (6-items) include day-to-day activities like housework or gardening. Total hours per week spent in both incidental and planned physical activity will be obtained by multiplying frequency scores and duration scores. Summation of the incidental and planned physical activity hours per week will also provide a total activity score. The IPAQ has been shown to have good test-retest reliability and concurrent and face validity [47]. The IPAQ is relatively short and easy to complete by older individuals and has been used previously in studies of fall risk factors and prevention programs in older people [48–50].
  - *The falls efficacy scale (Short FES-I)* questionnaire will be used to record fear of falling [51]. The FES-I consists of 7 items using a Likert scale that assesses the participant’s level of concern regarding the possibility of falling when performing certain daily activities. Items are scored from *1* = not concerned at all to *4* = very concerned. The total score ranges from 7 (not concerned) to 28 (Severely concerned to match to the description on score 7) where higher scores are associated to a greater fear of falling [51]. The test–retest reliability of the Short FES-I is good (*r* = 0.92) [51].
  - *Social activity participation* will be measured with a 10-item questionnaire which was derived from a measure of social functioning [52] and has been previously used to measure social participation in people who had repeated falls [53]. Participants will be asked to record the number of times in the previous two weeks that they have participated in 10 categories of social activities including: gone to church, visiting friends and family, gone to concerts, plays, or sporting events; gone to fairs, museums or exhibits; and attended meetings, appointments, classes/lectures. Questions use a five-value response option (less than once/week, once/week, twice/week, 3–6 times/week and every day). A summary score of social participation will be calculated as the total number of times in which the participant undertook any of the 10 activity categories during the period in question (two weeks). Higher scores are associated with a higher level of social activity.
  - Physical self-perceptions will be measured using the *Physical Self-Description Questionnaire (PSDQ) – Short Form* [54]. The PDSQ is a 40-item questionnaire scored from 1 (false) to 6 (true) and consists of 11 factors: Health, Coordination, Activity, Body fat, Sport, Global Physical, Appearance, Strength, Flexibility, Endurance and Global esteem. The PDSQ has been shown to have good test-retest stability over a 3 month period (*r* = .81 to .94) strong factorial structure and discriminant and convergent validity [54].
  - *Falls and physical activity calendar*—Participants will be requested to record any falls and physical activity or exercise experienced using a monthly calendar for 12 months from the baseline assessment. At the end of each month the calendar will be returned to the researchers in a reply paid envelope. If the calendar is not returned within two weeks of the end of a month, the participant will be followed up with a phone call. For this study, a fall will be defined as “inadvertently coming to rest on the ground, floor or other lower level, excluding intentional change in position to rest in furniture, wall or other objects” [14] and this definition will be explained to the participant to make sure they fill in the calendars accurately.

### Qualitative data

Participants allocated to EPIG will be interviewed by an experienced qualitative researcher at the end of the intervention period (18 weeks). An interview guide (see Additional file 1 – Interview Guide) will explore the participant’s experience with the project including reasons to volunteer to the project and their experiences with the training program (staff supervision, frequency, duration, progression of exercises, level of difficulty, changes to their life in general and general level of satisfaction). Only participants assigned to the exercise intervention group will be interviewed. In interviewing these participants, we aim to be able to identify the positive and negative elements of the exercise park program perceived by the participants, as well as the main participation barriers which can impact on the adherence and acceptability of the intervention. The interviews will be conducted on an individual basis by a researcher independent to the intervention. All interviews will be digitally recorded and transcribed verbatim. The interviews will be analysed using a thematic analysis approach [55]. Data will first be coded to identify and label text to the participants experience of the exercise park using both an inductive and deductive approach. These codes will then be placed into overarching themes. Inter-rater reliability will be examined by an independent coder on all of the themes and subthemes by reviewing a random sample of 10 % of all the excerpts relating to each theme and sub-theme with any differences in coding discussed between the coders [56].

### Feasibility

As the senior exercise park initiative is a new concept to the Australian older community, feasibility will be assessed and will be defined as the number of participants recruited and retained over the recruitment period, overall adherence and seasonal adherence, safety and adverse effects and number of sessions cancelled due to unfavourable weather conditions. In addition, the qualitative data collected via interviews of EPIG participants will be taken into account for feasibility purposes as they might more clearly show participants’ perceptions of this kind of initiative.

Overall adherence to the exercise program will be defined by the number of sessions attended: 100 % adherence if participant attended 35 sessions or 9 sessions of social meetings. EPIG or CG participants’ participation and attendance will be recorded via a spreadsheet diary and will be collected respectively by the exercise supervisor of that participant on each specific session or by the principal researcher. Physical activity calendars will be used to monitor if EPIG or CG participants have participated in any other physical activities during their participation in the study. Reasons for participants missing sessions will be documented on the spreadsheet diary. Participants will be given a phone call in case they miss two consecutive sessions without any communication with any exercise supervisors.

Considering that the exercise sessions are held outdoors, this study intends to investigate if the participants’ adherence would be influenced by weather conditions during the four seasons in Melbourne. Seasonal adherence will be recoded as adherence over Summer (December to end of February), Fall (March to end of May), Winter (June to end of August) and Spring (September to end of November). Also, the number of sessions that were cancelled due to rainy, windy and excessively hot days (above 37 °C) will be recorded given that these conditions would potentially put participant’s safety and health in risk.

Safety and adverse effects will be measured by the number of falls incidents that occur during exercise sessions, and will be recorded via an incident report form (treatment needed post-incident and related lesions or injuries). The circumstances surrounding the fall (e.g. muscle fatigue, dizziness) will be recorded. EPIG participants will be also asked in the following session (48 h) to report if they experienced any uncomfortable delayed muscle soreness or fatigue post-exercise that limited them from doing their daily tasks such as ascending and descending stairs, rising from a chair, and carrying shopping bags. The following question will be used: “Did you experience any muscle soreness after the session that limited you from doing your normal daily activities such as carrying shopping bags, rising from a chair or putting a t-shirt on?”. If they answer “yes” the muscle soreness event will be recorded on the participant’s spreadsheet diary.

### Community partner organizations

An important aspect of projects of this nature is the identification of the community partner organizations that can help with personnel, infrastructure and logistical matters needed for its successful running. A key element of the design of this project is to conduct it in the community and therefore create a better platform for research translation. Therefore, a number of community organizations with a focus on older people’s health promotion and specialised care were approached. These community organizations were mainly selected based on the nature of the work they have been involved in with this specific population group. Two community organisations: Catholic Homes and Gateway Social Support Options have partnered to collaborate in this research project. Catholic Homes provided the infrastructure and land for the equipment installation and allow for the exercise session to be conducted in the community setting. Gateway Social Support Options is a community-based organisation with over 200 older people living in the western suburbs of Melbourne. Gateway Social Support Options will provide access to its members for participant recruitment.

### Exercise park

**Fig. 2:**
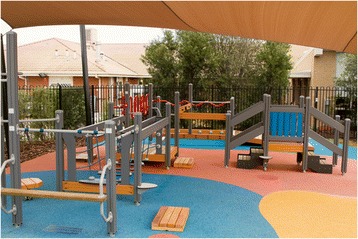
Lappset’s exercise park for senior population

The senior exercise park used on this project was provided in-kind by Lappset (Fig. 2) and it was installed at the St Bernadette’s Community Respite House in Sunshine North. The exercise park consists of a number of components and stations that aim to work on the following aspects of physical performance: upper body mobility and fine motor skills, balance and coordination, lower limb and upper limb strength, stretching and flexibility (Tables 1, 2, 3, 4 and 5).

### Familiarisation and exercise intensity

A familiarisation session will be organised for each participant prior to commencement of the exercise program. The exercises will follow the guidelines of the Australian Position Statement of exercise for falls prevention [57]. Participants will be introduced to the 10-point Borg Rating of Perceived Effort (RPE) scale [58] at their familiarization session.

The initial level of the exercises difficulty will be tailored to the capabilities of the participant with the primary consideration of safety. Adjustment of the exercises (i.e. increase in intensity and difficulty) will be made based on the participant individual progression. RPE will be used to determine the intensity of each exercise where participants will be encouraged to exercise with a RPE between 4 and 7/10. New exercises will be gradually introduced to the participants every 1–2 weeks (See Table 7). A participant will progress to the next level of exercise when an RPE of below 4/10 (‘too easy’) will be reported (Tables 1, 2, 3 and 4).

**Table 7:** Order of progression and introduction of new exercises for the 18 weeks of intervention

Week number	Exercise 1
1 and 2	Stations 1 to 5
3 and 4	Stations 1 to 6
5 and 6	Stations 1 to 7
7-18	Stations 1 to 8

### Individual and group exercise progression

Each station will include two exercises and will be performed twice by each participant. Two participants will be allocated in each station such that each participant will perform one at a time and will swap over (See Table 6). However, a participant can be exercising only with their exercise supervisor in case there is an uneven number. Participants will have different pairs and exercise supervisors on each session to stimulate social interaction. Participants will be given a resting period of 30–60 s which will be adjusted according to program progression as detailed in Table 8. The duration of each exercise will also increase based on program progression (Table 8). Rest periods between exercises will be used to discuss about difficulties and to provide further feedback. Participants will be allowed to have as many breaks as necessary to keep them performing the exercises with good technique and proper form. As some exercises such as step-ups on platform, ramp + walking through a net, and taps onto a platform can be more challenging to some participants due to the platform height, wooden blocks (L 70 cm x W 40 cm x H 10 cm) will initially be used for these participants. The same blocks will be used to make some exercises such as push-ups, pull-ups and sit to stand a bit more challenging to participants (Fig. 2).

**Table 8:** Set time and rest for exercise progression for the 18 weeks of intervention

Week number	Set time	Rest time
1 to 2	60 s	60 s to change over and rest
3 to 8	60 s	30 s change over and rest
9 to 14	75 s	30 s change over and rest
15 to 18	90 s	30 s change over and rest

### Fidelity monitoring

To assure that the core elements that contribute to the success of intervention studies will be correctly documented and can be successfully translated into community settings, a number of process measures will be used to document, track and enhance the fidelity of this project. Firstly, all exercise supervisors will receive a written exercise protocol manual to be followed in all sessions. Secondly, participants will have their attended sessions recorded by the supervising researcher to make sure that they are receiving nearly the same amount of exercise prescription at the end of the 18 weeks of exercise intervention. Participants who have missed more than eight sessions (for being unwell or away) at the end of the 18-week period will be asked to make up these missed sessions until they reach a minimum of 35 h (out of 50 expected hours) of exercise delivery. Finally, fidelity to treatment delivery will be monitored via the use of exercise cards (per participant) for the specific training date with further details of the participant’s performance and general suggestions for the next session (i.e. improve level of certain exercise, monitor pain, watch technique, etc.).

### Sample size

Power analysis was undertaken using previously published discharge data on the primary outcome measure - the BOOMER balance measure [26], and assuming an improvement of 3 points (reported as the minimum detectable difference [26]) and an effect size of 0.5. A sample size of 48 participants per group will be required for a power of 0.80 and alpha of 0.05. We will allow for 20 % dropout rate (this is a conservative estimate based on previous exercise programs with older people), and therefore will recruit 60 participants per group.

### Data management and statistical analysis

All analyses will be conducted on an intention-to-treat basis. If there are chance imbalances in baseline participants’ characteristics, then these variables will be used as covariates. Mixed linear modelling incorporating intervention and control groups at the baseline and two follow up time points (18 weeks and 26 weeks after intervention commencement), with the number of sessions attended and baseline physical activity level as covariates, will be carried out using SPSS. Comparisons will be made for the primary outcome (BOOMER) and secondary outcomes (functional tasks, strength measures, gait parameters, health related quality of life measures, and psychosocial measures). Although the number of falls is being recorded over a period of 12 months after the commencement of participants, this study is not powered for falls outcomes. Regarding the qualitative data being collected, the interviews from EPIG participants will be analysed using a thematic analysis approach [55], as described above.

## Discussion

In this community based study we aim to evaluate the effectiveness of an exercise intervention using an exercise park specifically designed for older people in reducing the risk of falls and improving strength and balance. Whether such exercise parks have an impact on reducing the risk of falls, improving muscle strength and balance and quality-of-life is not yet known. As such this planned trial will be the first to provide evidence if the exercise park can improve physical health or psychological well-being. In addition, this study aims to describe this innovative exercise approach, and report its feasibility and the challenges associated with running this in a community setting.

Falls and related injuries are the leading cause of disability among older adults [1]. Physical activity, more specifically exercise, has been shown to be effective in preventing falls in older people [10]. However, data from the 2011–12 Australian Health Survey: Physical Activity report found that only 36.6 % of males and 38.8 % of females over 65 engage in sufficient physical activity [59]. Considering the 75 and over group, these figures are even more problematic with just one in three men and one in five women being sufficiently physically active [59]. Thus, finding falls prevention initiatives that increase levels of physical activity and factors that contribute to adherence such as the exercise park initiative is important to reduce their risk of falling and their rate of falls.

Exercise parks may offer a playful and enjoyable experience to their users which may increase compliance for participation in fall prevention programs. By providing a fun but physically challenging environment, this novel and unique concept may provide an alternative strategy to enhance physical activity levels in older individuals and consequently increase their health and ability to cope more effectively with the challenges faced in their daily life. Subscribing to this idea, a recent study recommended that physical activity sessions that focus on overall movement rather than structured exercise program might be more achievable for the older population group [60].

Community-based exercise programs that focus on health and wellness for physically inactive community-dwelling seniors have been shown to be effective in reducing feelings of loneliness and social isolation [61]. After people retire, they are more likely to stay at home alone, watching television and reading newspapers, and consequently become sedentary in their lifestyle [62, 63]. The senior exercise parks may provide an opportunity for seniors to socialize more, improve their quality of life and, their physical and mental health. This novel method could be an option for them to exercise their bodies and minds in an enjoyable way through strength, balance and coordination exercises as well as simple activities to support a variety of activities of daily living.

The type of environment on which exercise-based therapeutic interventions are offered is vital for their success [60]. A study with post-menopausal women showed that indoor activities have been associated with negative feelings such as frustration, anger, sadness and anxiety whereas outdoor programs have been associated with positive feelings such as happiness, joy and pleasure [64]. Contributing to these findings, outdoor exercises were shown to improve mood and self-esteem in older people, and seniors tend to attend more to the outdoor sessions compared to the indoor ones [60]. However, outdoor sessions such as the one proposed by the senior exercise park program can be dependent on climate and seasonal conditions which has the potential to influence adherence [65]. The planned project will explore how participation can be affected by seasonal conditions.

The proposed intervention will use task-specific exercises (e.g. step and stair climbing, walking on unstable and uneven surfaces, etc.) which can be easily translated to older adults’ activities of daily living. In addition, senior exercise parks can be installed outdoors or indoors in public places such as community centres and parks free of charge to the public. While an intervention of this nature might comprise some financial and logistic engagement of the local councils and community organizations, this initiative could potentially be a cost-effective way to engage older individuals in a more active and healthier lifestyle.

This study is evaluating not only quantitative data but also qualitative data through EPIG participant interviews. Therefore, more comprehensive information to assess the acceptability, likely barriers, facilitators to adherence and general experiences of this targeted group throughout participation in the project can be obtained and that may help in the analysis of the feasibility of the proposed exercise intervention [66].

It has been demonstrated that older people of lower socio-economic status have a higher rate of hospitalisation due to falls [67]. Specifically in Melbourne, the western and northern suburbs of Melbourne comprise one of the areas of lowest income and more socioeconomic disadvantage [68]. Therefore, this would be an important area to target future interventions to prevent falls in older populations and one of the reasons why the exercise park used in this project is installed in Sunshine North, a western suburb of Melbourne. However, the western side of Melbourne is also marked by the existence of many multicultural groups and ethnicities (high presence non-English speaking immigrants) which can potentially make recruitment and retention of participants more complicated due to cultural and language barriers. Low literacy levels, competing responsibilities and location of the testing site and intervention have been listed as reasons why recruitment in these areas of lower socio-economic status may face challenges [69]. Thus, the outcomes of this study will examine how these mentioned factors play a role and may affect the feasibility of this kind of initiative in these areas.

Community based falls prevention interventions supported by community organizations are important as they have the potential to be sustained. Our focus will be on the benefits to the individual and the opportunities to continue such a program beyond the duration of the project. In addition, this project will provide policy makers with empirical evidence of the effectiveness of an exercise park and the factors which might influence the implementation of such parks on a larger scale.

### Limitations

This study has a number of limitations. Firstly, it has been reported that some participants who do not receive their preferred treatment may experience “resentful demoralisation” [19], may not comply with the program structure proposed, may not report accurate responses on the follow-up appointments and may even drop out from the trial [70]. This may introduce some bias which could possibly affect the internal validity of the trial. However, because their preference is being recorded before the randomization occurs, this preference will be taken into account when analysing and interpreting the results. Secondly, due to budget limitations, this study will not be blinded where the principal researcher will be conducting the assessments, the randomization and the exercise intervention. However, despite these limitations, the results of this study will be able to provide an insight on how older adults will respond to this novel and unique senior exercise park. Furthermore, this study will report the possible health benefits and well-being improvements on older people when using this exercise park and will guide further larger research trials.

## Conclusion

The outcomes of this project will provide empirical evidence for the effectiveness of the use of the novel exercise park in the community and how its use can improve physical (e.g. strength and balance), psychological (e.g. fear or falling and self-perception) as well as psychosocial (e.g. increased social participation) aspects of older people's lives. In addition, this study will explore the barriers to participation and the acceptability and feasibility of the senior exercise park in the Australian older community as a mode of physical activity in older age.

## Additional file

Additional file 1:
**Interview guide for the participants from the intervention group (EPIG).**

## Abbreviations

BOOMER: Balance outcome measure for elder rehabilitation
CG: Control group
EPIG: Exercise park intervention group
IPAQ: The incidental and planned activity questionnaire
Short-FES-I: The falls efficacy scale
PSDQ: Physical self-description questionnaire
RCT: Randomised controlled trial
*SF-12v2™*: The short form (12) health survey
