# Supplementary material for: A novel dynamic exercise initiative for older people to improve health and well-being: study protocol for a randomised controlled trial
Source: BMC Geriatr. 2015 Jun 24;15:68. doi: 10.1186/s12877-015-0057-5 (PMC4477416; doi:10.1186/s12877-015-0057-5)
Supplement: Additional file 1: — Interview guide for the participants from the intervention group (EPIG). [file 12877_2015_57_MOESM1_ESM.docx]

**Interview Guide for Exit Interview of the Participants of EPIG**

**Introduction**

1. Welcome
2. Consent interview and audiotaping the interview.
3. Confirmation of confidentially of information provided.
4. Inform participants that they can withdraw at any time during the interview.
5. Thanking participant for attending the interview and tell them what the interview is about (learning more about their experiences within the program and improve current practice).

**Questions**

1. Why did you volunteer to be part of the research project?
2. Could you tell me about your experiences from engaging in the training program, good or bad? (the abcd questions you only ask if they haven’t spoken about it)
   1. What aspect of the project captured your imagination most?
   2. What was it about the training program that you really enjoyed?
   3. Are there aspects of the training program which you think could be done better?
   4. If any what difficulties did you experience from engaging in the training program (e.g., were there any barriers to participation? If you skipped sessions, what were the main reasons for this?)?
3. What did you think about the frequency of the program (too frequent, just right, not enough and reasons)?
4. Was the length of the sessions of exercise adequate for you (too long, too short, and reasons)?
5. Was the progression in the program suited to your needs?
6. Could you say anything on the role of the exercise leader and the supervision you received during participation in the program?
7. As a result of you participating in the program, have you noticed any changes to your daily life? If yes, could you please describe them to me?
8. What do you think it may have caused this change to your daily life?
9. Is the training program something you would like to continue to participate in the future? If yes/no please explain.
10. Would you suggest this program to others?
11. If you would describe the program to this person in a few sentences what would you say?
12. On a scale from one to ten with one not useful and 10 extremely useful how would you rate the training program?
13. Is there anything else that you would like to mention about the project that wasn’t asked here?
